# Clinicopathological manifestations of coexistent monoclonal immunoglobulin deposition disease and immunotactoid glomerulopathy

**DOI:** 10.3389/fmed.2022.911998

**Published:** 2022-08-25

**Authors:** Yina Wang, Yu Yan, Bao Dong, Wanzhong Zou, Xin Li, Chunying Shao, Lei Jiang, Mei Wang, Li Zuo

**Affiliations:** ^1^Department of Nephrology, Peking University People’s Hospital, Beijing, China; ^2^Department of Pathology, Peking University Health Science Center, Beijing, China; ^3^Electron Microscope Laboratory, Peking University People’s Hospital, Beijing, China

**Keywords:** monoclonal immunoglobulin deposition disease, immunotactoid glomerulopathy, pathology, glomerulonephritis, multiple myeloma, renal biopsy

## Abstract

Combination of monoclonal immunoglobulin deposition disease (MIDD) and immunotactoid glomerulopathy (ITG) is a rare form of monoclonal immunoglobulin (MIg)-associated renal disease. We retrospectively reviewed the native kidney biopsy specimens at Peking University People’s Hospital from 2011 to 2020. Five patients were diagnosed as MIDD + ITG. Their clinical and pathological characteristics were studied. The typical clinical features were nephritic syndrome and renal dysfunction with prominent anemia, but hematuria was mild. Unlike single MIDD and single ITG, on light microscopy, segmentally distributed mesangial nodular sclerosis on the basis of mesangial matrix hyperplasia was the major lesion. Others including membranoproliferative glomerulonephritis (MPGN)-like lesion, glomerular basement membrane thickness, and mild to moderate mesangial and endothelial proliferations might presented at the same time and in the same glomeruli. On immunofluorescence, MIg, usually monoclonal light chains, deposited along glomerular basement membranes and tubular basement membranes, while the intact MIg or monoclonal heavy chain deposited in the mesangial regions. Corresponding to the depositions on immunofluorescence, punctate “powdery” deposits along glomerular basement membranes and tubular basement membranes under electronic microscopy indicated the presence of MIDD. Microtubular substructures (diameters of 20–50 nm) exhibiting hollow cores arranged in parallel arrays in mesangial regions indicated the presence of ITG. Patients treated with bortezomib-based regimen seemed to have better outcomes. In conclusion, MIDD + ITG is a rare combination form of MIg-associated renal disease. Accurate diagnosis requires the comprehensive pathological investigations.

## Introduction

Monoclonal immunoglobulin (MIg)-associated renal disease has heterogeneous morphologic forms (1). It usually presented in one form (2). Some of literatures reported combinations with two or more different pathologic forms (3–5). The most common pathologic form is monoclonal immunoglobulin deposition disease (MIDD) coexisting with light chain cast nephropathy (LCCN) (4, 6–9). However, the combination of two forms of glomerular diseases, especially the co-deposition of organized and non-organized structures was rare (3, 10). The presentation of this combination was not simply the add-on, but had unique characteristics. Up to date, no case of MIDD + ITG was reported. Here, we report the clinicopathological features, treatments and outcomes of 5 patients with MIDD + ITG, and help to understand the characteristics of this pattern.

## Materials and methods

All 11,767 native kidney biopsy specimens from 2011 to 2020 at Peking University People’s Hospital were reviewed from patients’ medical records. Patients fulfilled the diagnostic criteria of both MIDD and immunotactoid glomerulopathy (ITG) were enrolled in the study. MIDD and ITG were diagnosed according to previous literatures (2, 11, 12). Briefly, the patients were diagnosed as MIDD and ITG when there were typical depositions on EM (“powdery” deposits along basement membrane for MIDD and microtubular substructure exhibiting hollow cores arranged in parallel arrays for ITG) with IF proved MIg deposition.

Demographic and clinical information including age, gender, clinical symptoms, past histories of hypertension and diabetes, blood pressure, hemoglobin, urinalysis, urine protein output, serum albumin, serum creatinine were collected at the time of biopsy. MIg was detected by serum and/or urine immunofixation electrophoresis (IEF) and free light chains (FLCs) (Freelite, Binding Site, United Kingdom) test. Treatment and follow-up data were also obtained from the patients’ medical records.

All kidney biopsy samples were processed for light microscopy (LM), immunofluorescence (IF) and electronic microscopy (EM) examination using standard techniques. IF was performed on cryosections (5 μm) using polyclonal fluorescein isothiocyanate (FITC)-conjugated antibodies against IgG, IgM, IgA, C3, C1q, κ and λ light chains (Dako, Denmark), respectively. Determination of the IgG subclasses was performed using monoclonal FITC-conjugated antibodies to IgG1, IgG2, IgG3, and IgG4 (SouthernBiotech, United States). For LM, kidney biopsy specimens were stained with hematoxylin and eosin, periodic acid-Schiff (PAS), Masson’s trichrome, periodic acid-silver methenamine, respectively. Also, Congo red and immunohistochemical (IHC) staining (CD38, CD 138, CD3, and CD20) were performed. Ultrastructural evaluation was performed using a transmission electron microscope (Thermo Scientific, TECNAI SPIRIT, United States).

This research was carried out in accordance with International ethical guidelines for biomedical research involving human subjects (CIOMS) and Helsinki Declaration. This research was approved by the Ethics Committee of Peking University People’s Hospital (2121PHB-84-001). Informed consent was obtained from all participants.

### Statistical analysis

Continuous variables with normal distribution were expressed as mean ± *SD* and variables with non-normal distribution were expressed as median (Q_25_, Q_75_). Categorical variables were expressed as numbers or percentages.

## Results

### Patient characteristics

The incidence of MIDD + ITG was quite low, accounting for only 0.04% of biopsied patients in our center. Five patients were identified. The demographic characteristics are shown in Table 1. There were three males (60%) and two females with an average age of 61.0 ± 4.6 years at kidney biopsy.

**TABLE 1:** Demographics and clinical characteristics.

	Patient 1	Patient 2	Patient 3	Patient 4	Patient 5
Gender/age	F/68	F/61	M/58	M/56	M/62
Initial symptoms	Weight loss	Fatigue	Foamy urine	Foamy urine	Dark urine
Time from initial symptom to kidney biopsy (months)	6	24	18	6	6
Hypertension	N	N	Y	Y	Y
Edema	N	N	Y	N	N
Hepatosplenomegaly	Y	N	N	N	N
Other manifestation	N	N	N	N	Osteolysis
Hb (g/L)	87	86	80	80	92
Urine RBC/μl	38	70	27	556	43
Proteinuria (g/d)	0.53	0.93	1.28	3.15	3.38
Alb (g/L)	43.4	34.3	34.6	28.4	29.6
Scr (μmol/L)	142	101	206	656	137
SIFE/UIFE	IgA κ	Neg	κ	IgA λ	IgG κ
Serum FLC ratio (κ/λ)	NA	322.5/38.3 (8.39)	NA	52.2/179.25 (0.29)	136/13.5 (10.07)
Hematologic condition	MM	MGRS	MGRS	MGRS	MM

SIFE, serum immunofixation electrophoresis; UIFE, urine immunofixation electrophoresis; FLC, free light chain; NA, not applicable; MM, multiple myeloma; MGRS, monoclonal gammopathy of renal significance; N, no; Y, yes.

### Clinical characteristics

The clinical characteristics of the five patients are shown in Table 1. Foamy urine, dark urine, fatigue and weight loss were the main initial symptoms. The median duration from onset to diagnosis was 6 (6, 21) months (range 6–24 months). Microscopic hematuria was seen in all patients. Proteinuria level was 1.85 ± 1.32 g/d. Serum albumin was 34.1 ± 5.9 g/L. Four patients suffered renal insufficiency as chronic kidney disease (CKD) in three and acute kidney injury (AKI) in one. Patient 4 was dialysis-dependent before renal biopsy. Only one patient (patient 2) had normal renal function. The median serum creatinine level was 142 (range 101–656) μmol/L. Three patients (60%) had hypertension, and none had diabetes.

As shown in Table 1, two patients fulfilled the established diagnostic criteria for MM and three were monoclonal gammopathy of renal significance (MGRS). All patients had prominent anemia with hemoglobin level of 85.0 ± 5.1 g/L. Serum and/or urine IEF confirmed the presence of MIg in four patients except patient 2. She was proved to have MIg for the high κ/λ ratio of 8.39, despite the negative results of serum and urine IEF. The complement levels (C3 and C4) were normal in all the five patients. The results of autoantibodies, hepatitis B and C, and serum cryoglobulin were all negative.

### Pathologic findings

**FIGURE 1:**
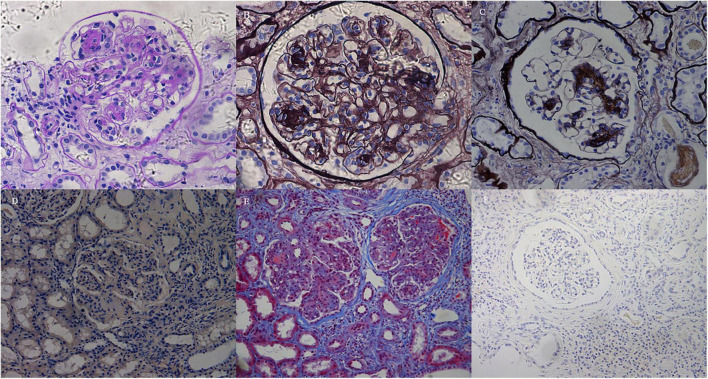
Light microscopy findings. **(A)** The glomerulus exhibited mesangial expansion and extensive proliferation of mesangial cells and matrix, with mesangial nodular sclerosis. The nodules and tubular basement membranes were periodic acid–Schiff (PAS) positive (Patient 3, PAS; × 200). **(B)** Diffuse membranoproliferative-like features revealed GBM duplication and mesangial interposition with double-contour or multicontour appearances (Patient 3, PASM; × 400). **(C)** The glomerulus exhibited segmental nodular sclerosis with mild mesangial hypercellularity. The GBMs in non-sclerotic areas were not thickened (Patient 4, PASM; × 200). **(D)** Congo red staining was negative (Patient 2, Congo red staining; × 100). **(E)** The mesangium expanded with diffuse hypercellularity and mesangial matrix proliferation. There was tubular atrophy and multifocal infiltration of lymphocytes and monocytes in the interstitium with fibrosis (Patient 2, Masson; × 100). **(F)** Lymphocytes and monocytes infiltrating the interstitium were CD38-negative by immunohistochemical staining (Patient 3, CD38; × 100).

The pathologic findings are shown in Table 2 and Figure 1. Mesangial nodular sclerosis was the major lesion on LM usually distributing segmentally, formed on the basis of mesangial matrix hyperplasia lesions. There were also mesangial and segmental endocapillary proliferation. Some manifested membranoproliferative glomerulonephritis (MPGN)-like lesions due to mesangial interposition resulted in double-contour or multicontour. However, the lesion only distributed focally and segmentally and the GBMs were thickened mainly at the site of double-contour or multicontour. The GBMs in the non-sclerotic area were not thickened. Congo red staining were negative.

**TABLE 2:** Renal pathological findings.

	Patient 1	Patient 2	Patient 3	Patient 4	Patient 5
Pathologic diagnosis	HCDD + ITG	LCDD + ITG	LCDD + ITG + ATIN	LCDD + ITG	LCDD + ITG
LM					
Major lesion patterns	MNS, MsHP	MNS, MPGN	MsHP, MPGN	MNS, MsHP	MNS, MsHP
IF					
Heavy chains	α++, GBM/TBM (linear), MG (coarse granular)	γ1++, MG (coarse granular)	α++, MG (coarse granular)	α++, MG (coarse granular)	γ2++, MG (coarse granular)
Light chains	Neg	κ+, GBM/TBM (linear), MG (coarse granular)	κ++, GBM/TBM (linear), MG (coarse granular)	λ++, GBM/TBM (linear), MG (coarse granular)	κ++, GBM/TBM (linear), MG (coarse granular)
EM					
Powdery electron dense deposits	GBM, TBM	GBM, TBM	GBM, TBM	GBM, TBM	GBM, TBM
Microtubular deposits	MG, Sub-Endo	MG	MG	MG	MG, Sub-Endo

HCDD, heavy chain deposition disease; LCDD, light chain deposition disease; ITG, immunotactoid glomerulopathy; ATIN, acute tubular-interstitial nephropathy; LM, light microscopy; MsHP, mesangial hyperplasia; MPGN, membranoproliferative glomerulonephritis; MNS, mesangial nodular sclerosis; IF, immunofluorescence; EM, electron microscopy; GBM, glomerular basement membrane; TBM, tubular basement membrane; MG, mesangium; Sub-Endo, subendothelial.

Except for the glomerular lesions, there were various degrees of tubulointerstitial injury, with tubular atrophy and multifocal inflammatory infiltration. Lymphocytes (CD3^+^ or CD20^+^) and monocytes were seen without eosinophils according to the HE staining. In IHC staining, CD38^+^ or CD138^+^ cells were rarely seen indicating no plasma cells infiltration (shown in Figure 1). No PAS-negative casts were found in the tubules.

**FIGURE 2:**
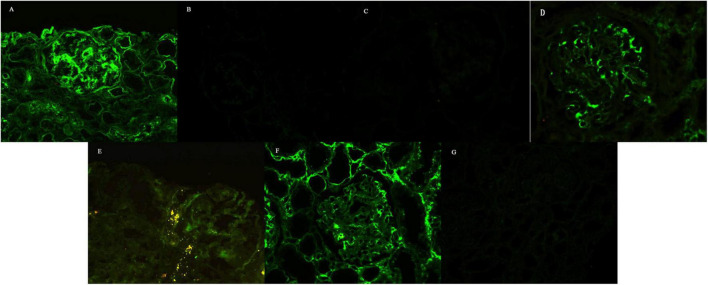
Immunofluorescence findings. **(A)** IgA linearly deposited along the GBMs and segmentally along the TBMs, while it was coarsely granularly deposited in the mesangial regions, with 3 + intensity (Patient 1, ×100). Staining for κ-chain **(B)** and λ-chain **(C)** was negative (Patient 1, ×100). Staining for IgG **(D)** and IgG2 **(E)** revealed coarse granular deposition only in the mesangial regions, without deposition along GBMs and TBMs (Patient 5, × 200). Restrictive κ-chain **(F)** was linearly deposited along the GBMs, segmentally deposited along the TBMs, and granularly deposited in the mesangial regions, while staining for λ-chain **(G)** was negative (Patient 5, × 100).

On IF, immune depositions with monoclonal light chain and/or heavy chain were consistent with the type of serum/urine MIg (Table 2). Monoclonal heavy chain without light chain was found in one patient, while the deposits of monoclonal light chain + heavy chain were found in other four patients. κ chain was the major type of light chain, while α and γ chains were the major types of heavy chains. In all the patients, light chains or heavy chain α deposited in mesangial region, along the GBMs and segmentally along the TBMs. Heavy chains were found mainly in mesangial region without deposition along GBMs and TBMs in four patients besides patient 1 (shown in Figure 2).

**FIGURE 3:**
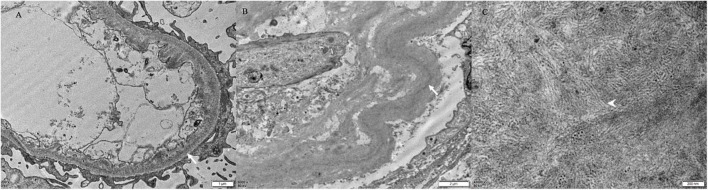
Electron microscopy findings. There are finely punctate “powdery” electron-dense deposits (white arrow) involving the inner aspect of the GBM **(A)** and the outer aspect of the TBM [white arrow, **(B)**] [Patient 4, **(A)** × 200, **(B)** × 6,000]. **(C)** The mesangial deposits comprised microtubular substructures (diameters of 20–50 nm) with hollow cores (arrowhead) arranged at least focally in parallel arrays (Patient 4 × 43,000).

Punctate “powdery” electron-dense deposits were seen in mesangial regions and along the GBMs and/or TBMs on EM. Concurrently, coarse granular deposits were found in the mesangial regions with a microtubular substructure (diameters of 20–50 nm) exhibiting hollow cores arranged in parallel arrays (shown in Table 2 and Figures 2, 3).

### Treatment and outcomes

Follow-up data were available for four patients (except patient 1) with a median duration of 31 (20, 54) months (range 18–60 months). Patients 2 and 5 received bortezomib-based therapy with or without autologous stem cell transplantation. They achieved complete remission of hematological and renal symptoms with improved renal function. Patient 3 was treated with steroids combined with cyclophosphamide and lenalidomide. Four months later, he advanced to end stage renal failure albeit hematological complete remission was achieved. After 60 months of follow-up, he remained dialysis-dependent. Patient 4 refused any chemotherapy and was treated with hemodialysis. After 18 months of follow-up, he also remained dialysis-dependent.

## Discussion

Although the incidence of single MIDD or ITG patients was relatively low (6, 13, 14), here we described a rarer series of patients with MIDD + ITG. Their characteristic pathological features had great diagnostic value, despite the non-specific clinical presentations. Segmental mesangial nodular sclerosis on the basis of mesangial matrix hyperplasia was the main lesion. MPGN-like lesion, GBM thickness and mild to moderate mesangial and endothelial proliferations presented at the same time and in the same glomeruli. EM manifestations of “powdery” deposits along GBM and/or TBM, and microtubular substructures with hollow cores arranged in parallel arrays in mesangial regions indicated the presence of both MIDD and ITG. At the same time, IF proved MIg depositions.

### Clinical features of MIDD + ITG

The patients in this study were all in the middle aged or older. It was similar to that previously reported in single MIDD or ITG patients (6, 13, 14). However, due to the small sample size in this study, we could not determine whether there was a gender difference in MIDD + ITG. The duration from onset to diagnosis was relatively long due to atypical early symptoms and late renal biopsy. Nephritic syndrome with chronic renal dysfunction was the prominent presentation of MIDD + ITG, in accordance with most MIg-associated renal diseases. The remarkable anemia incompatible with the patients’ renal function indicated the possible existence of hematologic diseases. The results of serum/urine IEF and/or FLCs implied the possible diagnosis of MIg associated disease. Our study was consistent with the reports in the literature that not all the patients have positive M-spike on serum/urine IEF, and serum FLCs assay can make up for this deficiency (6, 12, 15, 16).

The prognosis of these patients was heterogeneous. Similar with previous studies (17, 18), patients treated with bortezomib-based regimen seemed to have better outcomes. Further study containing more patients with longer follow up is needed to confirm this hypothesis.

### Unique pathological features of MIDD + ITG in contrast with each of monoclonal immunoglobulin deposition disease or immunotactoid glomerulopathy

As the non-specificity of the clinical manifestations, the accurate diagnosis relied on renal pathology. The manifestations on LM displayed the heterogeneity of lesions including mesangial nodular sclerosis forming on the basis of mesangial matrix hyperplasia, MPGN-like lesions, GBM thickness and etc. This was a unique feature of MIDD + ITG. Although mesangial nodular sclerosis could be seen in both single MIDD and single ITG, there were prominent differences compared with that in MIDD + ITG (6, 11, 19). Mesangial nodular sclerosis distributed focally and segmentally in MIDD + ITG in contrast to the diffusely and globally distribution in single MIDD. This was consistent with the literature reports that the combination of LCDD with other forms of disease had lower presence of nodular sclerosis and was only diagnosed through the powdery deposits on EM (20). Considering ITG, although mesangial nodular sclerosis might present in the late stage, mesangial and endothelial proliferation leading to MPGN-like lesions were the typical pathological features (12, 14, 21–23). However, in MIDD + ITG, less proliferation and only segmental MPGN-like lesions were seen. At the same time, the partial thickened GBMs resulted from mesangial interposition with a double-contour or multicontour appearance was more comparable with the feature of ITG, rather than that of MIDD mainly presenting as diffuse thickness of GBMs. So, we speculated that ITG might play more roles in the formation of glomerular lesions in our patients. At the same time, due to the non-homogeneous distribution of mesangial nodular sclerosis lesions and negative result of Congo red staining, amyloidosis could be excluded.

On EM, there were characteristic features to differentiate the components of MIDD + ITG. The punctate “powdery” deposits along the GBMs and/or TBMs was the feature of MIDD, while the microtubular deposits with hollow cores in mesangial region indicated the presence of ITG (6, 15, 23). Although cryoglobulinemic glomerulonephritis (Cryo GN) also produce microtubular structures, there were several differences to ITG. In ITG, the microtubules were relatively thicker than Cryo GN. On cross-section, microtubules of Cryo GN have 8–12 spokes emanating from the perimeter, making the cross-sectional outer diameter about 33 nm (24). No EM features relating to Cryo GN with the negative results of serum cryoglobulin have excluded the diagnosis. Thus, the coexistence of both “powdery” deposits along the GBMs and/or TBMs and microtubular deposits with hollow cores in mesangial region confirmed the diagnosis when there was evidence of MIg deposition in the kidney.

On IF, κ was the major light chain in patients with MIDD + ITG, consisting with the immune type of single MIDD and single ITG (2, 6, 13–15). γ and α heavy chains were both commonly seen, while γ1 and γ2 were the main γ subclasses. This was in accordance with the immune deposition types of ITG in the literature (13). The features of MIg deposition indicated the existence of different forms of the disease. In this cohort, heavy chains mainly deposited in mesangial regions, and light chains deposited along GBMs and TBMs, as well as in mesangial regions. This suggested that intact MIg might be the component of ITG deposits while restricted light chains were the component of MIDD. Thus, the four patients except patient 1 were diagnosed as LCDD + ITG. For patient 1, α heavy chain participated in both MIDD and ITG deposits indicating the diagnosis of HCDD + ITG.

Various degrees of tubular interstitial lesions incompatible with glomerular lesions were also visible in four patients except patient 2 in this study. These might explain the renal dysfunction presented in the patients. Pathologically, according to IHC results, the infiltrated inflammatory cells were mainly CD3^+^ T cells and CD20^+^ B cells. Together with manifestations of routine staining, there was no evidence of allergic nephritis, LCCN, myeloma infiltration, or any tubulointerstitial damage caused by MM or MGRS. Considering the significant immune deposition along TBMs, renal dysfunction presented either chronically or acutely in patients with MIDD + ITG, may be related to the involvement of renal tubules and interstitium of MIDD. Taken together, careful and thorough investigations with the combined application of IF, EM, LM and IHC could provide an accurate diagnosis of MIDD + ITG.

### Possible mechanisms of MIDD + ITG

We reported a rare MIg-associated renal disease in which MIg deposited in both organized and non-organized ultrastructures. According to the pathogenesis reported (10), MIDD and ITG might resulted from immunoglobulins (MIgs) of different origins with unusual or abnormal structures. The deposition may be the consequence of the acquired defects in podocyte functions relating the clearance of the filtrated and retained immunoglobulin, which created the unique environment for deposition (19, 25). According to the immune phenotype, we hypothesize that in MIDD + ITG, intact MIgs with abnormal structures might result in organized deposits. At the same time, light chains or heavy chains were more likely to deposit on GBM, due to the abnormal physicochemical properties of MIg. We presumed that this might be one of the reasons to cause the acquired defects in podocyte functions. These defects promoted the retaining and deposition of organized intact MIgs with abnormal structures, leading to the formation of MIDD + ITG.

At the same time, whether the organized and non-organized deposits resulted from the same MIg of different conformations remained to be elucidated. In the series of LCDD + AL reported by Said, through the method of laser microdissection-assisted liquid chromatography-tandem mass spectrometry (LC-MS/MS), the combination was thought to be caused by pathological light chains produced by subclones stemming from one immunoglobulin light chain rearrangement, with a distinct mutated complementary determining region. Despite the lack of LC-MS/MS exam, we speculated that MIDD + ITG in this study was also caused by the same MIg for the following reasons. Firstly, MIDD and ITG deposits on IF had the same light chain or heavy chain isotype. Secondly, none of the patients had two different MIgs in the serum or urine. However, the accurate evidence needs to be obtained from LC-MS/MS.

In this study, the extraordinary combination of MIDD + ITG were described. The major limitation of the current study was that because of the limited number of cases, it was difficult to accurately summarize the prognostic factors for the outcomes. More cases are needed to further confirm our findings and speculations.

## Conclusion

MIDD + ITG is a rare form of MIg-associated renal disease. Clinically it mainly presents as nephritic syndrome and renal dysfunction with prominent anemia. Serum/urine IEF or serum FLCs may prove the existence of MIg. The accurate diagnosis relies on the comprehensive pathologic investigations. Larger cohort would help to determine the best choice of treatment of MIDD + ITG. The accurate evidence of whether the coexisted organized and non-organized deposits came from the same MIg, needs to be obtained from LC-MS/MS.

## Data availability statement

The original contributions presented in this study are included in the article/supplementary material, further inquiries can be directed to the corresponding author.

## Ethics statement

The studies involving human participants were reviewed and approved by the Ethics Committee of Peking University People’s Hospital. The patients/participants provided their written informed consent to participate in this study.

## Author contributions

YW, YY, and BD concepted and designed the study. WZ, XL, CS, and LJ analyzed and interpreted the data. YW and YY drafted the article and revised the article. MW and LZ provided intellectual content of critical importance to the work described. YY final approved the version to be published. All authors contributed to the article and approved the submitted version.
